# Application of rapid evaporative ionization mass spectrometry in preclinical and clinical analyses of steatotic liver tissues and cells

**DOI:** 10.1038/s41598-025-93305-w

**Published:** 2025-03-18

**Authors:** Julian Connor Eckel, Lena Seidemann, Mohamed Albadry, Gerda Schicht, Marija Skvoznikova, Sandra Nickel, René Hänsel, Daniel Seehofer, Grit Gesine Ruth Hiller, Hans-Michael Tautenhahn, Uta Dahmen, Georg Damm

**Affiliations:** 1 Department of Hepatobiliary Surgery and Visceral Transplantation, Clinic for Visceral, Transplant, Thoracic and Vascular Surgery, Leipzig University Medical Center, Leipzig, 04103 Germany; 2 Saxonian Incubator for Clinical Translation (SIKT), Leipzig University, Leipzig, 04103 Germany; 3 Department of General, Visceral and Vascular Surgery, Experimental Transplantation Surgery, University Hospital Jena, Jena, 07747 Germany; 4 Department of Pathology, Faculty of Veterinary Medicine, Menoufia University, Shebin Elkom, Menoufia 6131567 Egypt; 5 Department of General, Visceral and Vascular Surgery, University Hospital Jena, Jena, 07747 Germany; 6 Institute for Medical Informatics, Statistics and Epidemiology (IMISE), Leipzig University, Leipzig, 04107 Germany; 7 Institute for Pathology, Leipzig University Medical Center, Leipzig, 04103 Germany; 8 Comprehensive Cancer Center Central Germany (CCCG), Jena and Leipzig, Germany

**Keywords:** Rapid evaporative ionization mass spectrometry, MASLD, Lipidomics, Steatosis, Lipids, Biomarkers, Translational research, Biomarkers, Medical research, Bioinformatics, Mass spectrometry, Statistical methods

## Abstract

Rapid evaporative ionization mass spectrometry (REIMS) shows promise as a preparation-free tissue analysis tool with the prospect for real-time diagnostics. Given that hepatic steatosis is characterized by shifts in lipid species and abundance, we selected it as basis for method development, as REIMS specifically measures lipidomic profiles. However, further validation and protocol refinement are necessary to establish its clinical utility. In this study, we applied REIMS to steatotic human liver tissues, focusing on its ability to differentiate varying degrees of steatosis. We established standardized protocols for tissue handling and lipid analysis, which were essential for reliable data interpretation. Notably, our findings revealed that tissue size impacts REIMS sensitivity, with smaller samples yielding lower total ion counts and altered lipid profiles. Through principal component analysis, we identified key lipid classes, namely triacylglycerides, fatty acids, and glycerophospholipids. Despite a missing link between triacylglyceride abundance and degree of steatosis, we successfully identified condition-specific lipid patterns, with ceramides emerging as markers of advanced steatosis. Our study provides a protocol for the measurements of lipid standards showing the detailed degradation of specific lipids using iKnife-coupled REIMS. It highlights the pitfalls and limitations and provides critical recommendations for REIMS use. It also emphasizes the need for standardized biobanking and tissue preparation to ensure accurate lipid profiling, laying the groundwork for future protocol adjustments required for clinical application.

## Introduction

The term metabolic dysfunction-associated steatotic liver disease (MASLD) refers to a spectrum of liver diseases that are characterized by the excess storage of intracellular lipids together with one metabolic risk factor (e.g. type 2 diabetes mellitus or obesity)^1^. With the increasing prevalence of obesity and metabolic syndrome worldwide, MASLD has become the most common liver disease globally and is the fastest growing cause of primary liver cancer and liver transplantation^2,3^. Since the dysregulation of lipid metabolism plays a major role in the onset and progression of many inflammatory and malignant diseases, lipidomic profiling has come into focus^4^. In lipidomics, liquid chromatography-mass spectrometry (LC–MS) has become a widely adopted technique, appreciated for its precision, high throughput, and comprehensive lipid profiling capabilities^5,6^. However, liver biopsy and histopathological evaluation of hepatic lipid content remain the gold standard for diagnosing and staging MASLD. Although lipidomics has significantly advanced and has contributed to our understanding of MASLD pathophysiology, it has not found its way into routine diagnostics^7^. The complexity and time required for lipidomic analysis, along with the lack of clinical translation of lipid profile alterations from biopsies have hindered its implementation into clinical practice^8^.

The rapid evaporative ionization mass spectrometry (REIMS) technology has been developed to achieve near real time tissue diagnostics based on the mass spectrometric analysis of the smoke resulting from electrosurgical tissue dissection^9,10^. Biological tissue is vaporized with an electrosurgical knife and the resulting aerosols are fed into a time-of-flight detector allowing very precise mass measurements. Lipids are the most abundant species detected by this technique^11^. It has the potential to differentiate between different stages of disease and can be used to discriminate between tumor and non-tumor tissues based on their distinct lipidomic profiles^12^. It can furthermore support basic research efforts in identifying specific lipid species that account for the underlying pathomechanisms of MASLD. Comparison of lipidomes from classic LC–MS with iKnife-coupled REIMS shows that the latter does not reflect the cellular lipids qualitatively and quantitatively^12,13^. LC–MS can employ both targeted and untargeted approaches, where a wide variety of compounds are separated chromatographically and analyzed with high sensitivity, making it ideal for precise quantification and identification in complex mixtures^14^. In contrast, REIMS follows a shotgun approach, directly ionizing the sample through thermal evaporation without prior separation, allowing for near-instantaneous, untargeted analysis^15^. However, it lacks the quantitative precision of LC–MS. LC–MS and REIMS could complement each other in future analyses, e.g. by rapidly identifying interesting samples in larger cohorts, which could then be analyzed in depth by LC–MS. The application of REIMS is influenced by pre-analytical conditions, such as technical, thermal, and sample conditions, as already investigated by other groups. Specifically, Lin et al.^16^ have focused on the influence of temperature, sample volume, and gas flow rates. Wang et al.^12^, who used iKnife-coupled REIMS, have investigated the conditions of the electrosurgical unit. The results of both groups indicate that standardized analytical conditions are a prerequisite for the effective use of REIMS.

**Fig. 1:**
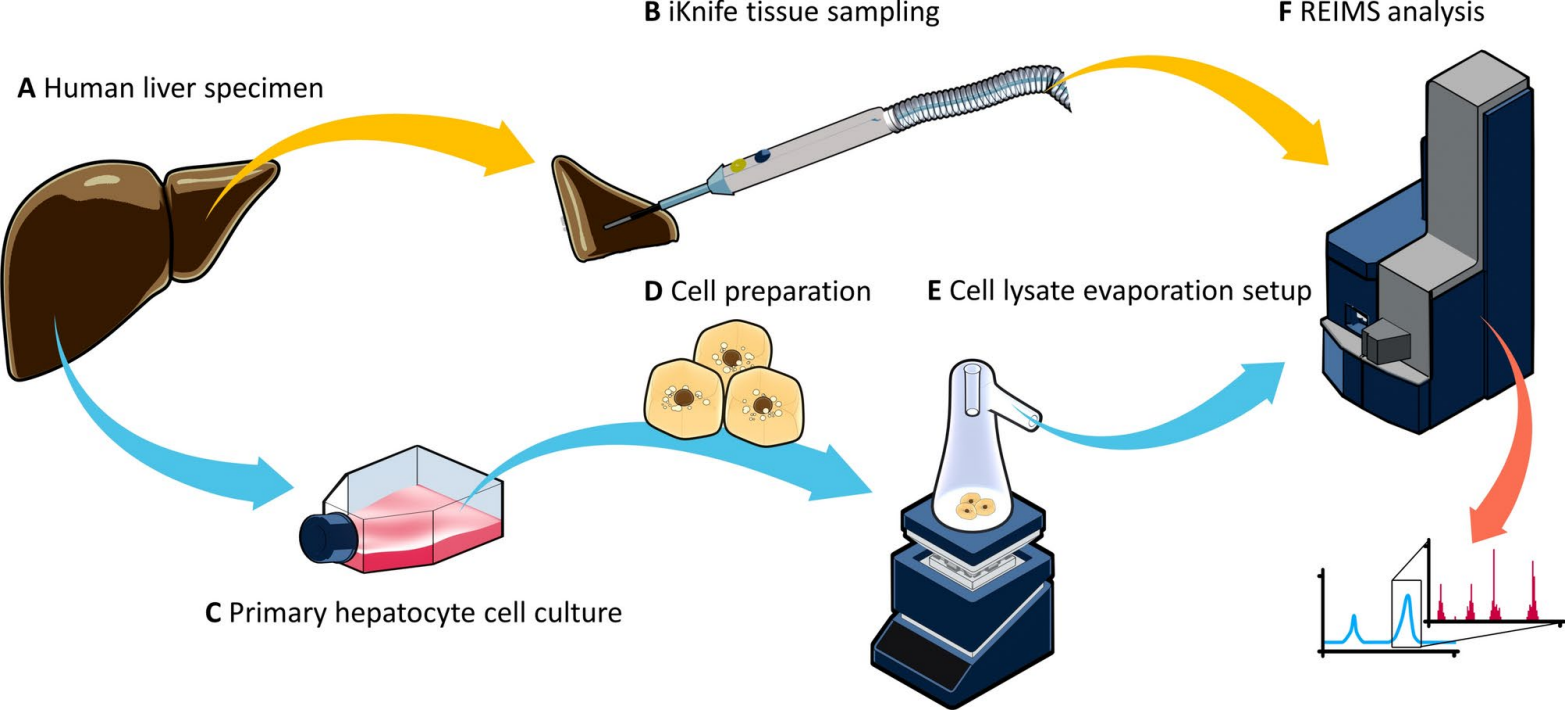
Experimental Setup of REIMS analyses. Liver tissues were collected during surgery (**A**). Samples from healthy and steatotic human and mouse livers were vaporized with an electrosurgical scalpel (iKnife) (**B**). Primary human hepatocytes (PHHs) were cultured and steatosis was induced with free fatty acids (**C**). Cells were harvested and lysed (**D**). Cell lysates were evaporized in a borosilicate glass flask, which was preheated on a Mini Hot Plate Preheater (**E**). Resulting vapors from both experiments were led into the mass spectrometer, afterwards chromatogram peaks were separated and the resulting spectra used for further analysis (**F**).

As a result, our goals were (1) to define standardized experimental conditions which better reflect the state of the tissue, and (2) to identify potential limitations in terms of e.g. certain lipids which are not reliably detectable. Consequently, we optimized the iKnife sample evaporation for REIMS and developed a new method for the standardized REIMS analysis of liquid samples (Fig. 1). We investigated the influence of sample conditions by analyzing cohorts from different clinical sites. Further, we used standardized in vitro and in vivo samples as well as reference compounds to investigate limitations in lipid detection. We used our results to optimize the REIMS analysis pipeline and give recommendations for sample standardization to improve lipid detection by iKnife-coupled REIMS.

## Results

### REIMS investigation of human steatotic liver samples from separate cohorts revealed a grouping depending on three different lipid classes

**Fig. 2:**
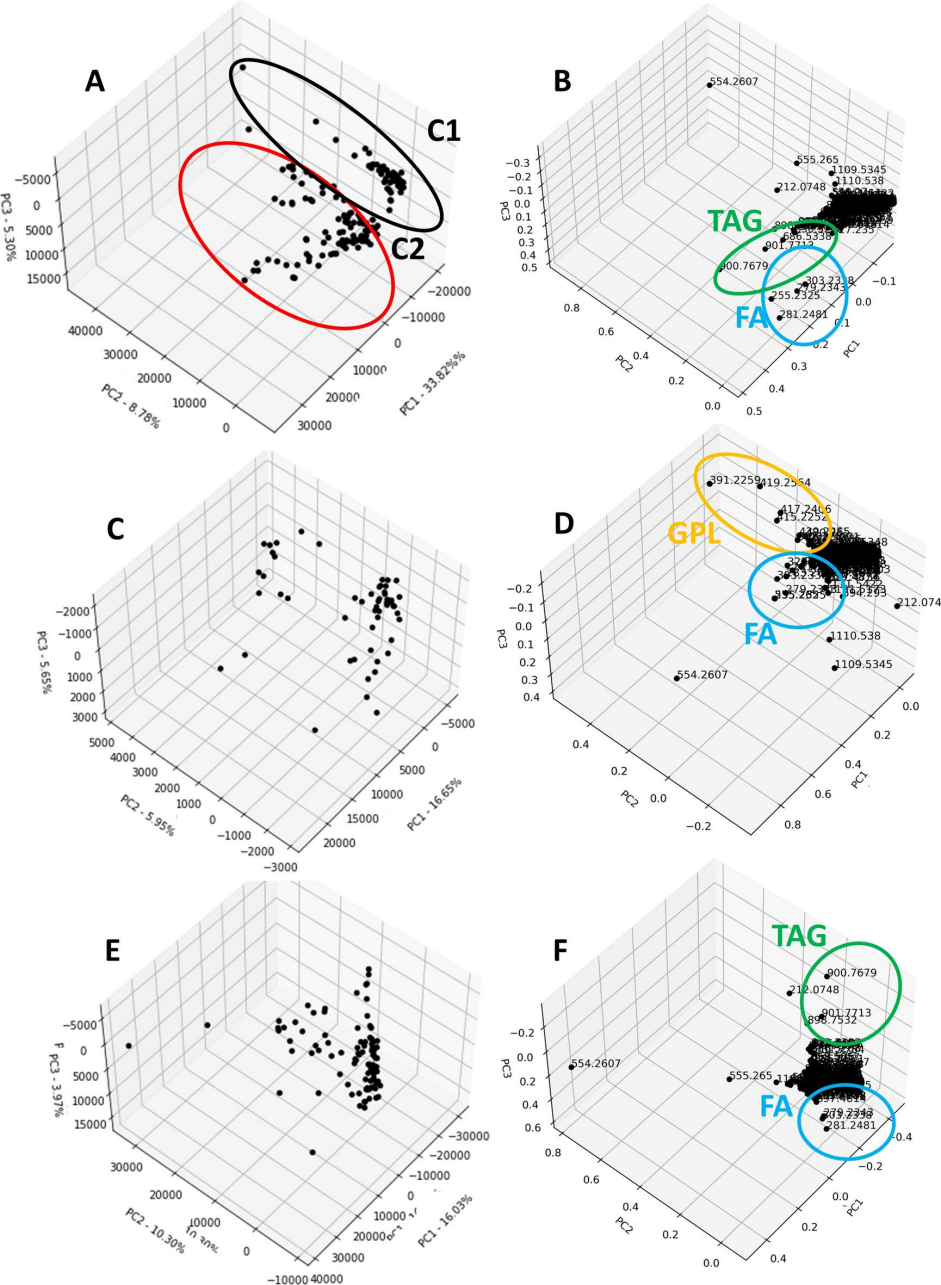
iKnife-coupled REIMS analysis of human liver tissue samples. Spectra were obtained from 39 human liver tissue samples collected at two different clinical sites. Each data point represents one measurement. Cohort 1 (C1) contains the results from 20 human liver samples which were measured in 3 technical replicates. Cohort 2 (C2) contains 19 human liver samples which were measured in 5 technical replicates. PCA of the combined data (**A**) and of the separated cohorts according to tissue source ((C1) **C**, (C2) **E**). Loadings of the combined data (**B**) and the separate cohorts ((C1) **D,** (C2) **F**). The loadings of the combined data consist of 1449 masses, *FA* fatty acids, *TAG* triacylglycerides, *GPL* glycerophospholipids.

iKnife-coupled REIMS analyses were performed on human liver tissue samples obtained from 39 patients who underwent liver resection at two German University Hospitals. A total of 1449 masses were identified in the combined samples. Unbiased bioinformatic analysis was performed. Data were plotted as principal component analysis (PCA; Fig. 2A), which revealed grouping into two clusters. Introduction of the clinical cohort labels into the data display revealed a near-perfect match with the formerly identified clusters. A test for variance homogeneity between the data sets generated from the two different tissue sources was performed using Levene’s test, which revealed significant heterogeneity (see Supplementary Table 1). Therefore, subsequent analysis was performed for the two separate cohorts (Fig. 2C, E). In the loading plots of the total sample (Fig. 2B) and the separated cohorts (Fig. 2D, F), lipids from the classes of triacylglycerides (TAGs), fatty acids (FAs) and glycerophospholipids (GPLs) were identified as major variables that determined PCA clustering. Since intracellular lipid storage is mainly made up of TAGs, an impact of the underlying degree of hepatic steatosis on REIMS tissue analysis was assumed^17^.

### Characterization of steatotic liver tissues revealed a characteristic lipid pattern

**Fig. 3:**
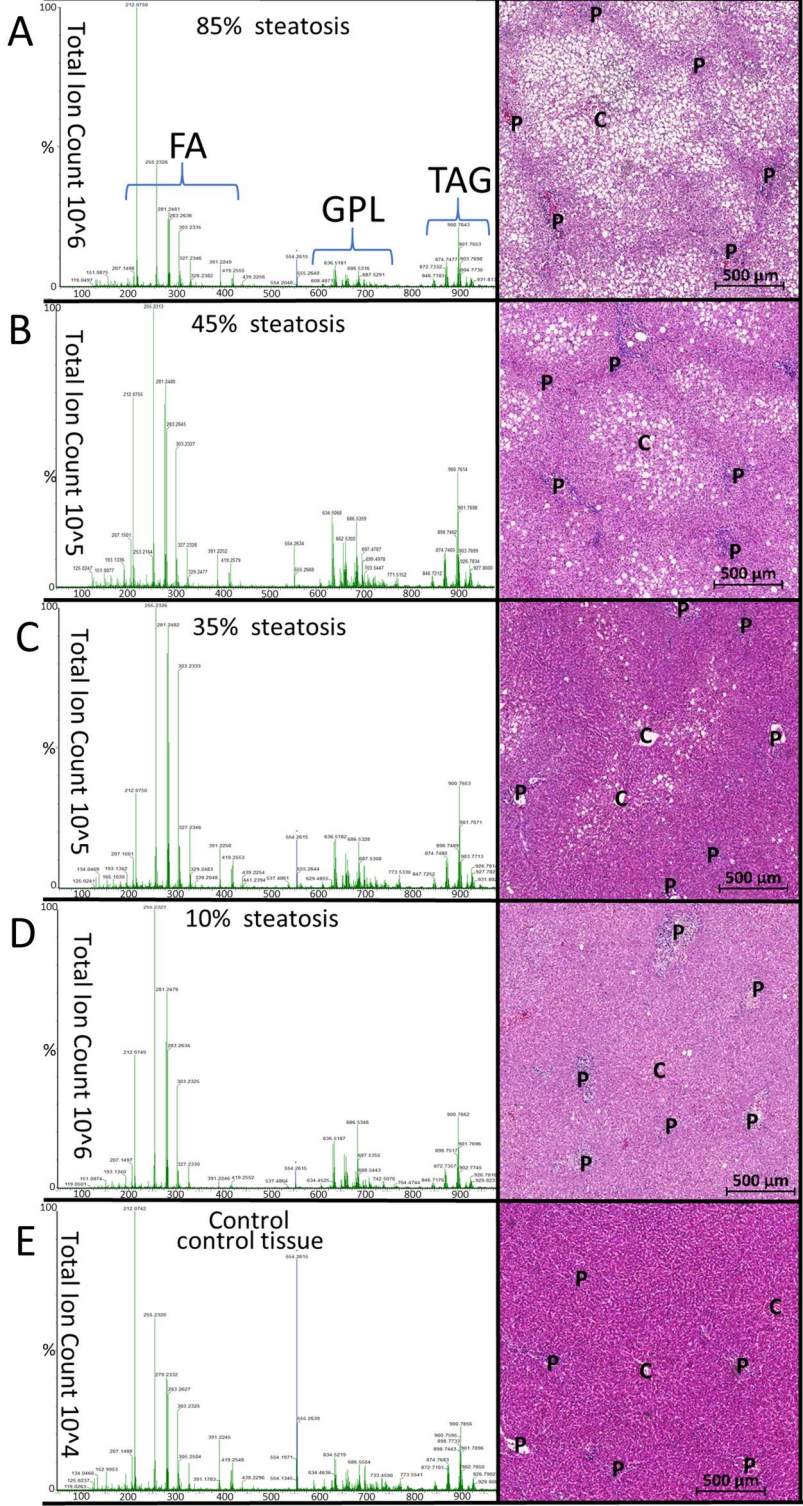
Spectra of steatotic human liver tissues obtained by iKnife-coupled REIMS and their corresponding HE- stained samples from the same donor (**A**–**E**). Mass spectra of steatotic liver tissues showed characteristic lipid patterns consisting of: *FA* fatty acids, *TAG* triacylglycerides, *GPL* glycerophospholipids. HE stainings show representative liver lobes (C, central vein; P, portal vein). White spots in the stainings are intracellular lipid droplets. HE stainings from all human liver tissue samples are provided in Supplementary Fig. 1.

Thus, we compared spectra of tissue samples with varying severities of steatosis, as the percentage of hepatocytes displaying micro- and macrovesicular steatosis (Fig. 3). Because of TAGs being the main intracellular lipid storage form, we expected a proportional increase in TAG intensity with increasing degrees of steatosis. In comparison to the TAG peak intensity, the increase in FA and GPL intensities should be less pronounced. However, we observed a rather similar increase of peak intensities over all lipid classes when comparing steatotic samples with control tissues (< 5%). The comparison of tissue samples with differing degrees of steatosis showed similar patterns with varying intensities in the FA and GPL lipid classes. The expected increase in TAGs proportional to steatosis was not observed. Thus, spectra generated with iKnife-coupled REIMS did not reflect the TAG quantities as they are actually stored in the liver tissue, even if they exceed 35% of the sample content.

### Validation of relevant lipids accounting for steatosis induction in vitro and in vivo

In a next step, we investigated hepatic steatosis under controlled conditions in established in vitro and in vivo models. Isolated primary human hepatocytes (PHHs) from 3 different donors were treated with 0.6 mM oleate and palmitate in a 2:1 ratio for up to 48 h. Defined cell counts were measured by heating station (HS)-coupled REIMS under newly established standardized conditions that allowed quantitative evaporation and analysis. Cellular measurements using REIMS have been described before, but lacked quantitation of the input cell number^18^. We therefore designed a method allowing for constant heat application in a defined volume, in which we added the desired cell count.

**Fig. 4:**
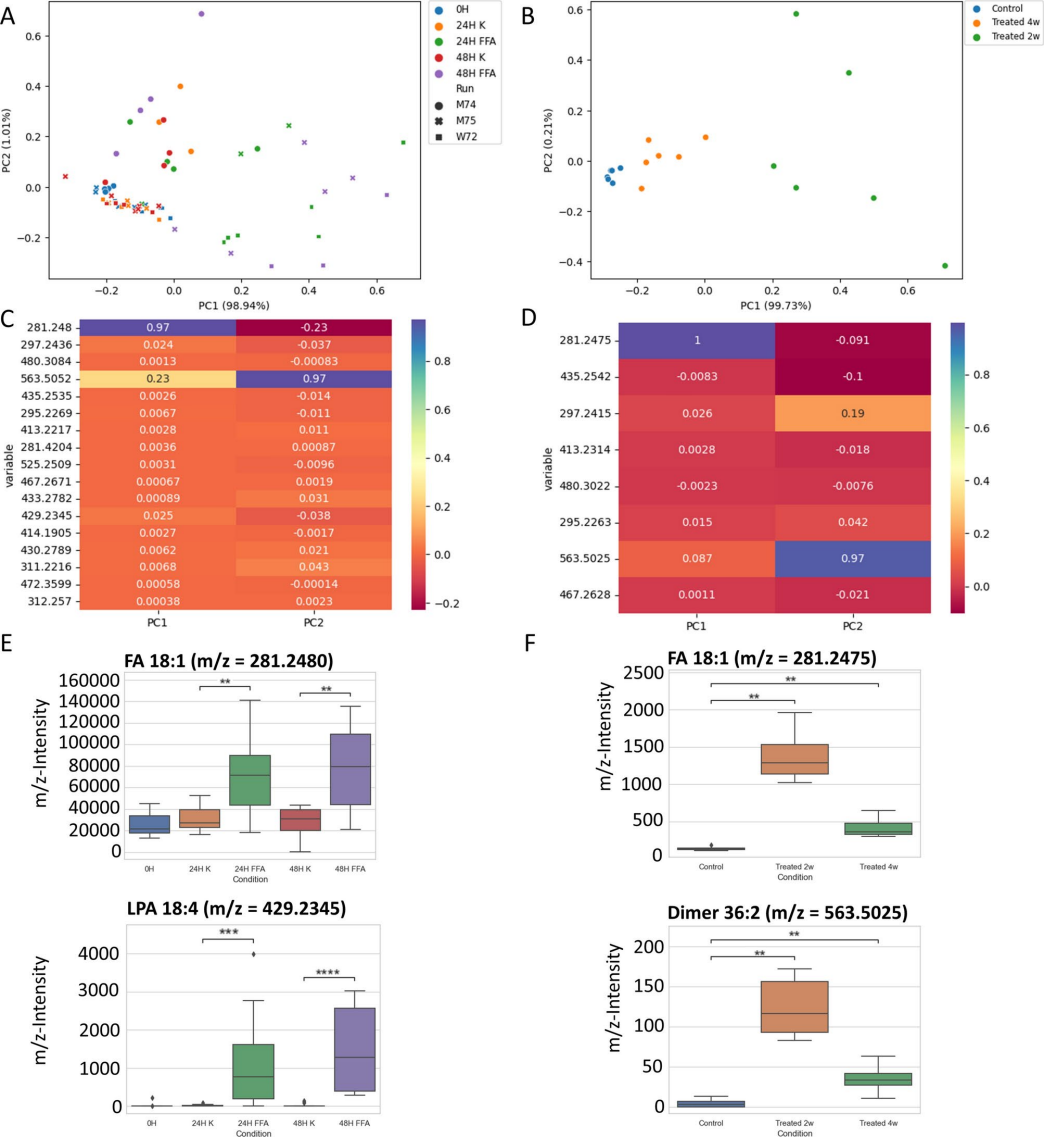
Investigation of lipids relevant for steatosis development in vitro and in vivo. PCA plot of lipids detected in primary human hepatocytes that were isolated from healthy control tissues and treated with 0.6 mM free fatty acids containing 1/3 palmitic and 2/3 oleic acid for 24 h (**A**). PCA plot of lipids detected in liver tissues from mice that were fed a high fat diet for 0, 2 and 4 weeks (**B**). (**C**,** D**) Loading heatmaps visualizing the contribution of each lipid to the first two principal components (PC) of (**A**,** C**), respectively. Median/quartile minimum/maximum box plots of two selected lipids from (**A**) (**E**). Median/quartile minimum/maximum box plots of two selected lipids from (**C**) (**F**). P-values (ns: p ≤ 1, *: 0.01 < p ≤ 0.05, **: 0.001 < p ≤ 0.01, ***: 0.0001 < p ≤ 0.001, ****: p ≤ 0.0001) were determined with Kruskal–Wallis ANOVA-type test. Further median/quartile minimum/maximum box plots were provided in Supplementary Fig. 2. The assignment of identifications to determined m/z was carried out using LipidMAPS. *K* control, *FFA* free fatty acids, *w* weeks, *M* male, *w* female.

For in vivo experiments, C57BL6/J mice were fed a high fat diet and liver samples were measured with iKnife-coupled REIMS after 0, 2 and 4 weeks. Steatosis-relevant lipid species were identified. Based on their standardized normalized abundance, 17 lipid species were selected as relevant for the in vitro experiment and 8 for the in vivo experiment (Fig. 4C,D). Subsequent PCA are displayed in Fig. 4. Measurements from steatotic PHHs shifted to the right in comparison to their respective controls (Fig. 4A). Although analyses were done under very standardized conditions, the single measurements still showed rather high variances, especially after the induction of steatosis. Control samples after 0 h from all 3 donors showed low variance. Also, the murine samples showed a shift to the right in PCA after steatotic treatment (Fig. 4B). Samples taken at different time points formed defined clusters. The loadings of the masses with the strongest influence in PCA are displayed as heatmaps (Fig. 4C, D). Notably, all relevant compounds identified from the in vivo experiment were also found in the in vitro measurements. Database-driven mass analysis using LIPID MAPS® suggests that some identified masses were oxidation and degradation products of oleic acid and oleic acid-containing lipids^19^. Statistical analysis with Kruskal–Wallis ANOVA-type test showed that oleic acid (m/z = 281.46) and its dimer with m/z = 563.50 were statistically significantly elevated in steatotic cells. This was not surprising, since oleic acid was the major component used for the induction of steatosis. However, the same lipid pattern was detected in the high fat diet-fed mice which underlines its relevance also in the (patho-) physiological development of steatosis (Fig. 4E, F).

### Identification of distinct lipid species and their fragments by iKnife-coupled REIMS

**Fig. 5:**
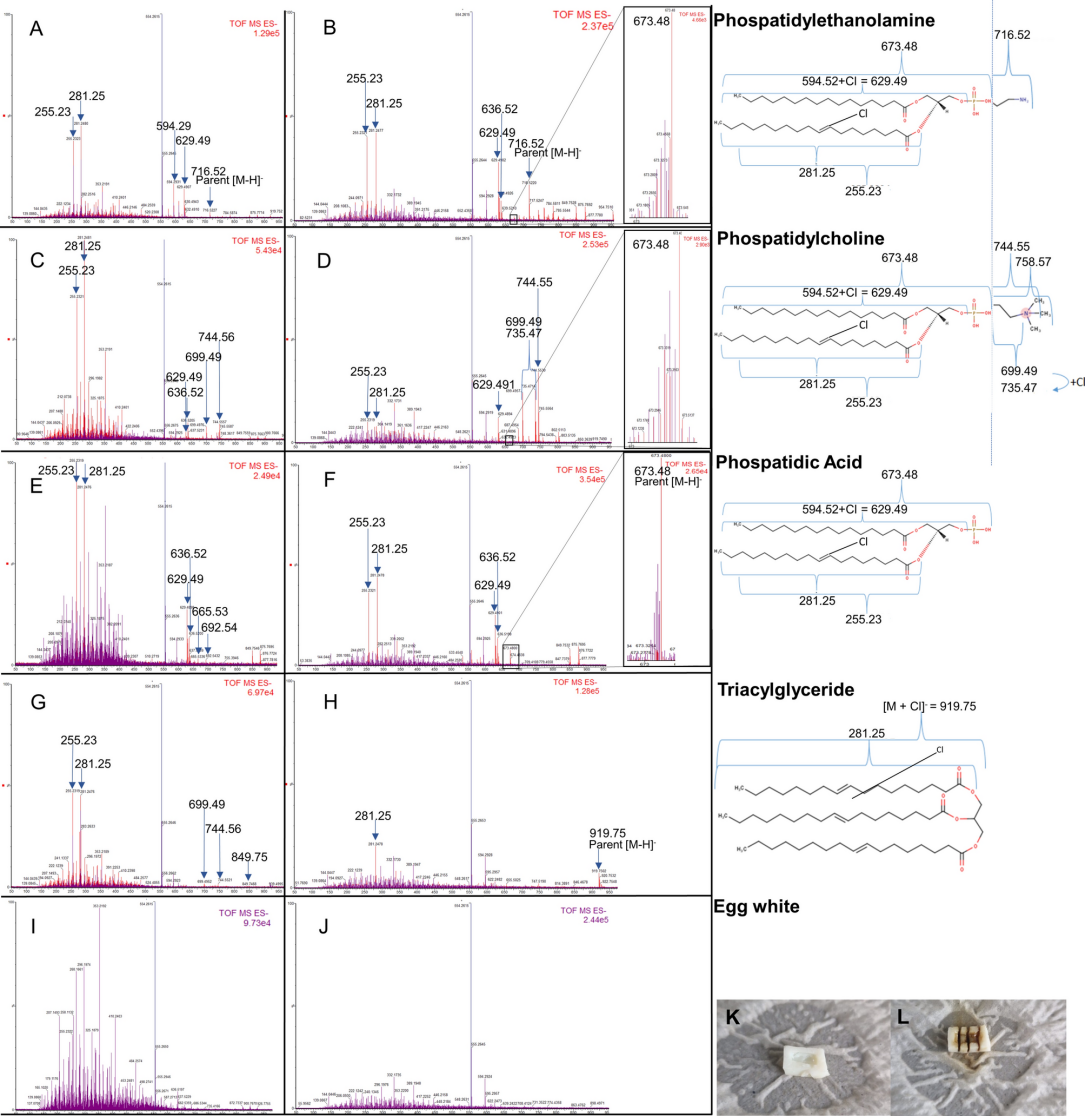
Detection of lipid standards by iKnife-coupled REIMS. Lipid standards were applied on an egg white matrix which was cut with the iKnife-coupled to REIMS. Spectra of samples treated with physiological saline solution are shown on the left side (**A**,** C**,** E**,** G**,** I**). Spectra of samples treated with saline solution and 0.1% formic acid are shown on the right side (**B**, **D**,** F**,** H**,** J**). Parent masses and fragments of interest are highlighted. (**K**, **L**) show examples of the egg white matrix before and after measurement. Further measurements of lipid standards are depicted in Supplementary Fig. 3.

In order to unambiguously identify lipid species and their fragments, commercially available lipid standards were purchased. In advance, we had searched the literature thoroughly, but did not find any publications reporting the measurement of lipid standards with iKnife-coupled REIMS. Because we wanted to investigate the influence of the iKnife on single lipid species and the resulting degradation products detected by MS, application of the HS-coupled REIMS did not meet our purpose. The challenge was to combine the dissolved lipid standard with a cuttable matrix. The new method we established, allowed for evaporation of the standards with the iKnife on a cuttable matrix of denatured egg white, which itself generates lipid spectra with low intensities (Fig. 5I, J). Analytical quality was improved by the addition of saline solution only or saline plus formic acid. Acidification led to stabilization of the parent of certain lipid species, e.g. phosphatidylethanolamine (PE; Fig. 5A, B) and phosphatidic acid (PA; Fig. 5E, F). The parent mass of PA was only detectable after acidification. Furthermore, PA was detected as a degradation product of PE and phosphatidylcholine (PC) after acidification. With regard to PC degradation Vaysse et al. used [M–CH_3_] as an adduct to find the sphingomyelin-PC parent^20^. The parent mass of PE was already detectable after the application of saline only but showed a higher peak intensity after the further addition of formic acid. In both cases, less fragmentation was also observed after acidification. Chlorination enabled the detection of PC (Fig. 5C, D) and of TAG in combination with formic acid (Fig. 5G, H). In general, it was observed that FAs were released from all lipid species, while diacylglycerides (DAGs) and PA were degradation products from GPLs rather than from TAGs. Taken together, these results show that the presence of ions and the pH value influences the detection of lipids in REIMS analysis. In addition, the results prove that some masses in iKnife-coupled REIMS spectra reflect degradation products.

### Optimization of mass identification and reduction of lipid species allowed the identification of influential masses in human steatotic liver tissues

In the next step, we aimed to optimize the bioinformatic analysis of iKnife-coupled REIMS spectra. A workflow was applied to the data set of steatotic liver tissue samples from the two German University Hospitals (see Fig. 2 and Supplementary Fig. 4). First, we assigned the individual samples to conditions according to their clinically reported lipid content (control (< 6, 6–20, 21–35, > 35%)). Then, data reduction was performed in Progenesis Qi by the exclusion of masses with a p-value ≥ 0.05. The mass range subjected to further analysis was defined as 250 to 1000 m/z. The resulting PCA showed a good clustering into the assigned steatosis conditions within the individual cohorts. Nevertheless, the two distinct cohorts exhibited minimal overlap (Supplementary Fig. 4G). Furthermore, the quantity and quality of identified masses differed between cohorts 1 and 2. The measurements for cohort 2 showed a higher quantity of masses and masses with larger m/z (Supplementary Fig. 1A, B). The primary distinction between the two sample sets was the size of the samples. Cohort 1 consisted of smaller samples which forced us to perform cuts closer to each other. An examination of the influence of the distance between two cuts revealed that the lipid patterns have a higher FA content with decreasing distance, while the proportions of GLP and TAG decrease in parallel (Supplementary Fig. 5). In summary, smaller sample sizes yielded lower total ion counts and altered lipid profiles.

**Fig. 6:**
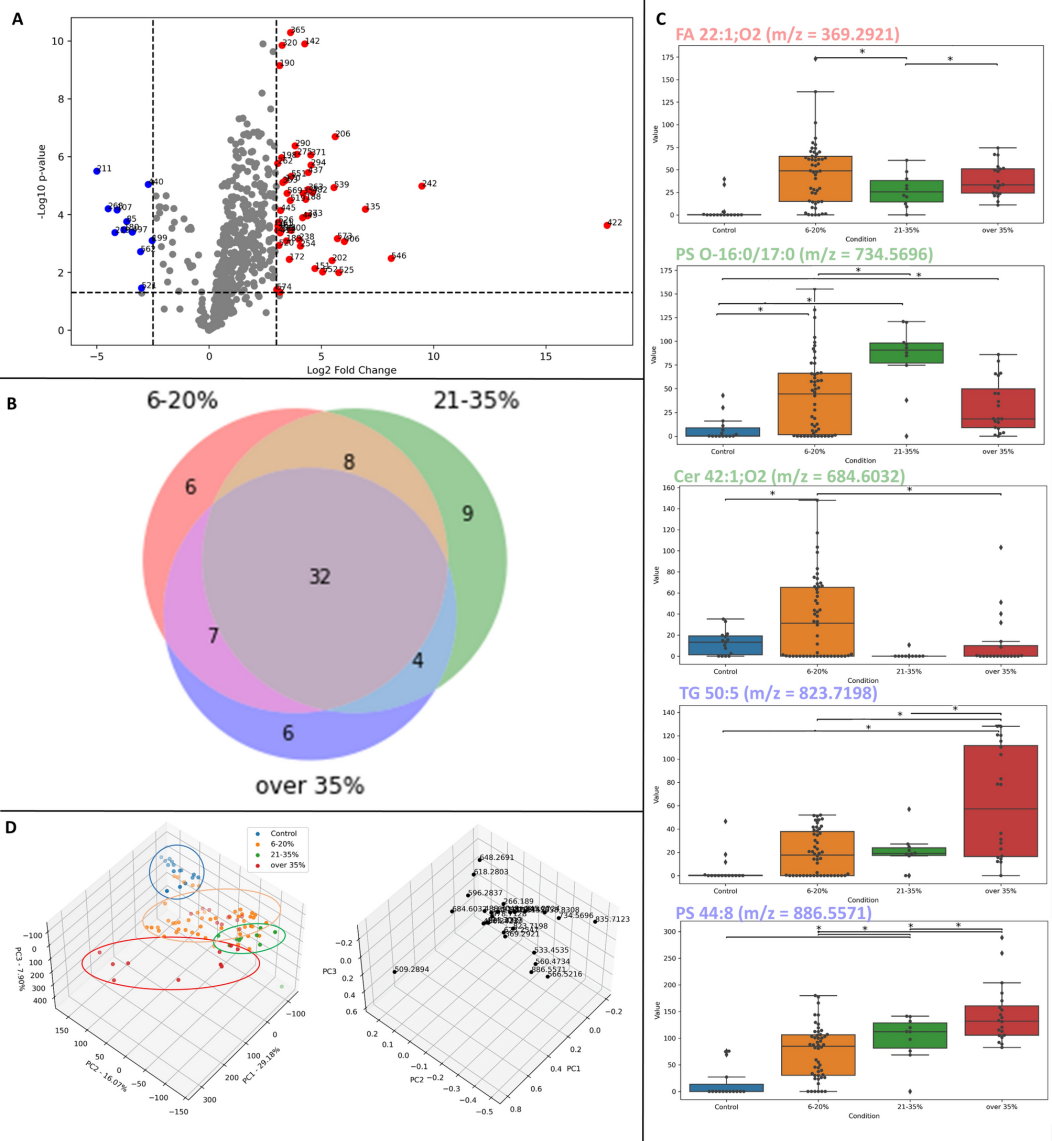
Optimized protocol of REIMS data analysis. A workflow for data reduction was applied to the data set of steatotic liver tissue samples from cohort 2 (see Fig. 2). Individual samples were assigned to conditions according to their clinically reported lipid content. Data reduction of the condition over 35% lipid content compared to the control using p-value and a threshold (decreased masses (blue), increased masses (red)) to identify relevant masses (**A**). Assignment of increased specific masses to the steatotic conditions (**B**). Representative masses specific for one steatotic condition were shown as box plots, (ns: not significant, *p <  = 0.05) (**C**). PCA plot and loadings of cohort 2 grouped by steatotic conditions after data were reduced to condition specific masses (**D**). The assignment of identifications to determined m/z was carried out using LipidMAPS. *FA* fatty acid, *PS* phospatidylserine, *TG* triacylglyceride

In the following, bioinformatic analysis was continued with the data of cohort 2 (Fig. 6). For further data reduction, we compared the steatosis groups to controls using an unpaired, two-sided *t*-test. Significantly increased and decreased masses with a log2 fold change of <  −2.5 or > 3 were defined as relevant (Fig. 6A and Supplementary Fig. 6). The number of thus identified masses in each of the steatotic conditions relative to control and to each other is visualized in Fig. 6B and C. There were no masses exclusively defining a steatotic state; neither compared to each other nor to the control (Fig. 6B and Supplementary Fig. 7). Instead, there was an overlap of relevant masses between the steatotic conditions (Fig. 6C and Supplementary Fig. 8). However, our data reduction led to a clustering of the cohort 2 data according to the steatotic conditions (Supplementary Fig. 7D). Twenty-six masses (21 increased, Fig. 6B and 5 decreased, Supplementary Figure 7A) were identified using LIPID MAPS® suggesting that ceramides were a lipid class defining the different steatotic conditions (Supplementary Fig. 9).

## Discussion

Since its introduction to the scientific community in 2010, REIMS has been utilized across various applications^16,21–24^. Scientists working with the REIMS technique have especially appreciated its simplicity in sample measurements^16,25^. Although probably not reaching the precision of LC–MS analyses, its rapidity in data acquisition has convinced many that it shows potential for future applications in clinical tissue diagnostics, provided its limitations are addressed through further protocol optimization and validation^14,20,22,26^. It has even been used in the operating theatre under study conditions^27,28^. Nonetheless, use of this technique is still restricted to research purposes, primarily with focus on discrimination of tissues of different origin^9,27^. Concerning tumorous tissue, the discrimination between metastases and harboring organ tissue is easier than the discrimination between primary cancers and their respective organs^20^. The identification of progressive disease stages in the same tissue type remains challenging^26^.

We investigated human liver tissue samples with different degrees of steatosis with the aim of identifying lipid patterns that describe the underlying pathomechanism of hepatic steatosis. In our study, we focused on specific lipid classes, including TAGs and GPLs, due to their well-documented roles in liver disease pathogenesis and progression. TAGs and their composition of FAs are key markers of lipid storage and are closely associated with hepatic steatosis. Their accumulation reflects imbalances in lipid metabolism, such as increased de novo lipogenesis or impaired lipid export^29^. Phospholipids, on the other hand, play crucial structural and functional roles in cellular membranes and are integral to processes like lipid transport, signaling, and bile formation. Alterations in phospholipid profiles have been linked to disruptions in membrane integrity, oxidative stress, endoplasmic stress, and inflammation—key contributors to liver disease progression from steatosis to steatohepatitis^30^. By analyzing these lipid classes, we aimed to assess the ability of REIMS to provide deeper insights into the molecular mechanisms of lipid accumulation and to identify potential steatosis-based biomarkers. In comparison to our approach, the previous study of Balog et al. analyzed a limited mass spectrum focusing on GPL^9^. Although we analyzed tissue samples with very high fat content, the expected increase in TAG in comparison to control tissues was less pronounced. Instead, the predominantly detected lipid class consisted of FA. These results were confirmed under the standardized induction of steatosis in vitro and in vivo experiments. The measurement of in vitro samples required the development of a new evaporation method, which was inspired by Lin et al.^16^ who used an electric heating probe to evaporate liquid samples. To achieve a more standardized set-up, we used a closed system, with a constant temperature and sample volume and a defined cell number. The in vitro treatment for steatosis induction consisted of a mixture of palmitic and oleic acid, but only oleic acid was identified as a marker lipid. Notably, oleic acid was also detected as the marker lipid in hepatic tissues of mice, where steatosis was induced by diet.

To investigate the observed discrepancy between TAG and FA detection, we aimed to study the influence of the iKnife on the various lipid classes. For that, a new method had to be developed to investigate the behavior of single lipids under conditions of iKnife-coupled REIMS. We tested different matrices in preliminary experiments and discovered that the process of lipid ionization by iKnife required a protein-containing matrix. An additional requirement for the matrix was a low lipid content to minimize unspecific measurements. Denatured egg white fulfilled both conditions and was used for measuring lipid standards. Further optimization of the detection method was achieved by the addition of ionization additives, as described previously. Sodium chloride was chosen because it facilitates better ion formation for TAG, thereby improving their detection. Formic acid, a well-known additive in LC–MS, was also employed to enhance ionization, ensuring more reliable and precise measurements^31,32^. Since oleic acid was the predominantly detected lipid in steatotic hepatocytes and murine hepatic tissues, we analyzed triolein. The detection of the triolein parent required acidification and chlorination. To a much larger extent, we measured degradation products, among which oleic acid was the most intense. Degradation products were similarly detected in the measurements of all other lipid standards. Thus, the spectra obtained by iKnife-coupled REIMS are highly influenced by sample preparation and the ionization method itself and do not directly reflect the biological lipidome of analyzed tissues.

Despite their artificial nature, degradation products are an integral part of the tissue fingerprint obtained by iKnife-coupled REIMS. We can define two classes of degradation products: (1) degradation products which also appear as biological compounds like phospatidic acid and (2) degradation products without a reference to cellular metabolites like [M-15]- ions of PCs. The first group are inadequate biomarkers because these interfere with their biological counterparts. However, group 2 are usable biomarkers because these are unique degradation products, especially when these are detectable in a higher abundance in comparison to their parent ions as seen in PCs. To allow better identification of degradation products in future REIMS studies, REIMS-specific data bases should be developed based on the systematic analyses of lipid standards.

Factors influencing the degradation process also have an influence on the acquired spectra. Wang et al.^12^ investigated different cutting conditions with iKnife-coupled REIMS in liver tissues. They showed that the signal intensities of lipids varied depending on the cutting mode and power. Their conclusion was that higher cutting power can generate more tissue aerosol, but too much power can lead to interference and thus reduce signal intensities of lipids^12^. In our initial analyses of liver tissues from two different cohorts, the major discriminant in mass detection was that cohort 1 delivered smaller total ion counts than cohort 2. The liver tissue samples from cohort 1 were smaller than those from cohort 2 and thus had a smaller contact surface with the neutral metal electrode. Cutting with the same electrical power therefore led to a higher energy density, visible by a greater heat damage and presumably leading to the observed reduction in signal intensities of the whole lipid spectra. Consequently, larger masses, which already show low signal intensities, vanish in the background noise. In line with this, the measurements of cohort 1 also miss relevant lipids from classes of GPLs and TAGs. This is critical because restriction to corresponding m/z range of GPL, as performed by Balog et al.^9^ and Mason et al.^26^, would result in highly varying lipid fingerprints for these samples and a PCA clustering rather on sample conditions than on their lipidome. Consequently, cluster differentiation of our data into different degrees of steatosis by PCA was only possible in the separated cohorts, but not for the combined data. This issue is particularly critical for analyses comparing tissues of varying sizes, especially in cases involving tumors and corresponding reference tissues, as tumor samples are often very small. Subsequent model development based on such data may distinguish tissues primarily by sample size rather than molecular characteristics, potentially fail in clinical translation. Thus, future studies should define standards for sample size and cutting distances in advance.

Improved bioinformatic analysis allowed a cluster separation into the defined steatotic conditions. Data reduction using statistical methods was a key to retrieve patterns of masses that define the steatotic state of the sample. Consequently, our bioinformatic workflow allowed the identification of relevant masses to describe increasing degrees of hepatic steatosis. A remarkable proportion of the thus identified masses were classified as ceramides (m/z = 340.2891; 510.4498; 560.4734; 566,5216; 684.6032) by LIPID MAPS®, which have been described as products of aberrant de novo lipogenesis in steatotic livers and exert lipotoxic effects^33^.

In our study, we demonstrate that REIMS provides a unique lipidomic profiling approach for characterizing hepatic steatosis, differing significantly from traditional histopathological methods. Histopathology relies on staining techniques to visually assess and semiquantitatively grade lipid droplets, offering valuable spatial information but lacking the ability to identify specific lipid classes or molecular alterations. In contrast, REIMS provides rapid, preparation-free lipidomic profiling, enabling the identification of key lipid classes such as TAGs, FAs, and GPLs, which are central to steatosis pathogenesis. Additionally, REIMS detected condition-specific lipid patterns, including ceramides associated with advanced steatosis and lipotoxicity, offering molecular insights beyond the scope of classic histological steatosis grading. The techniques complement each other in that our REIMS protocol could support model building to identify the degree of steatosis, comparable to histopathological grading.

Our study has certain limitations. The tissues we used were obtained from our biobank and thus fresh frozen. Further, our clinical tissue characterization and HE staining originate from the department of pathology, reflecting a different region of the resected tissue than the one we analyzed. Additionally, we did not perform cross-validation, such as a leave 20% out approach, to assess model robustness because our primary focus was on providing a descriptive analysis of the data set, rather than developing a predictive model. Our study aimed to explore and interpret the underlying structure of the data and assess group differences using statistical tests. Since we did not intend to create a model for feature identification or classification in an independent cohort, cross-validation was not applicable or necessary for the scope of this study. In the near future, it will be our goal to develop a machine learning model for our bioinformatic workflow that better assesses the conditions and disease states of liver tissues. This model will incorporate cross-validation techniques to increase the robustness of these conclusions. In this regard, lipid class-specific degradation products could act as potential biomarkers and help in standardizing and strengthening the REIMS technique. Fiorante et al. recently published a review containing recommendations for the use of ambient mass spectrometry data to create robust prediction models^34^. Our preliminary aim was to establish preanalytical standards for robust data acquisition to feed such models. Consequently, our data analysis was mainly in accordance with Fiorante et al. and complements the requested high standards for handheld ambient mass spectrometry analyses^34^.

Even though REIMS analysis is a technique which requires no sample preparation^25^, we showed that sample standardization is a prerequisite.

We learned from our analyses that:

- Sample size has an influence on analytical depths.
- Compounds that are difficult to be measured in the negative mode can be made detectable by ionization additives.
- Degradation products are an essential part of the REIMS lipidome and contribute to the tissue-specific fingerprint.
- REIMS does not directly reflect quantities within a biological lipidome of analyzed tissues.
- Quantitative deductions on the distribution of lipid species are only possible in cases of drastic biological differences.
- Data reduction is essential for the identification of relevant and influential lipid species.

In comparison to classic LC–MS-based lipidomics, REIMS analysis is limited by its disability to detect the comprehensive biological lipid spectrum and in delivering reliable quantitative measurements of lipids.

To make REIMS applicable in the clinical setting, awareness of these challenges is required. Results and models based on smaller tissue samples used in a laboratory setting cannot be directly transferred to an intraoperative setting or in clinical tissue diagnostics. Also, the use of ionization additives has to be adapted to the respective application. In surgery, saline is certainly applicable, but formic acid rather not. In pathological tissue diagnostics, both additives and even further ones (e.g. ammonium salts) may be used.

Thus, we recommend standardized biobanking strategies and sample collecting SOPs to guarantee sample comparability in terms of constitution and composition. Next, the preanalytical sample preparation has to be adapted to obtain a suitable lipid profile in relation to the research issue. This is a particular challenge when translating ex vivo established models into clinical applications. Given the challenges we faced in our REIMS experiments in differentiating lipid profiles for advanced clinical stages, clinical deployment of the technique will require further extensive protocol adjustments.

## Methods

### Collection of human liver tissue samples

Human liver tissues were obtained from patients undergoing liver resection at the University Hospitals of Leipzig and Jena, Germany. The study was conducted in accordance with the principles of the Declaration of Helsinki and approved by the Ethics Committees of the Medical Faculty of Leipzig University (registration number 322/17-ek, date 2020/06/10, ratified on 2021/11/30 and registration number 422/21-ek date 2021/11/10, and 450/21-ek date 2021/11/10) and of the University of Jena (registration number 2018-1246-Material). All patients gave their informed consent. Tissue samples were taken from macroscopically tumor-free liver tissues and either used for primary cell isolation or snap frozen and stored in liquid nitrogen until further use.

### Tissue characterization

HE-stained tissues were provided by the Institute of Pathology, Leipzig University Medical Center. Confocal microscopy imaging of HE staining was performed on an AxioScan Z1 at the Paul Flechsig Institute for Neuropathology and Brain Research. Characterization of the tissue, such as hepatic lipid content or tissue structure, was performed by the Institute of Pathology.

### Isolation and cultivation of human liver cells

PHHs were isolated from human liver tissue samples by a two-step EGTA/collagenase perfusion technique as described previously^35^. After isolation, the cell suspension was centrifuged at 51 × *g*, 5 min, 4℃ to separate PHHs from the NPC (non parenchymal cells) fraction. The PHH pellet was washed with phosphate buffered saline (DPBS; Gibco, Paisley, UK) and re-suspended in PHH culture medium (William’s Medium E with GlutaMAX™ (WME; Gibco, Paisley, UK), supplemented with 10% fetal bovine serum (FBS; Sigma-Aldrich, St. Louis, MO, USA), 15 mM HEPES, 1% nonessential amino acids (MEM NEAA 100x), 1 mM sodium pyruvate, 100 U/100 μM penicillin/streptomycin (all provided by Gibco, Paisley, UK), 32 U/L insulin (Eli Lilly and Company, Indianapolis, USA) and 1 μg/mL dexamethasone (JENAPHARM, Jena, Germany). Viable cells were counted with the trypan blue (Sigma-Aldrich, St. Louis, MO, USA) exclusion technique using a Neubauer counting chamber. PHHs were then either cultivated immediately for in vitro steatosis induction or cryopreserved. For cryopreservation, a PHH suspension of 5 × 10^6^ viable cells per ml PHH cell culture medium was centrifuged at 51 × *g* for 5 min at 4 °C and supernatant was removed. Cells were resuspended in an appropriate volume (maximum 1 × 10^7^ cells/mL) of hepatocyte cryopreservation medium (containing 70% ChillProtec® plus-Medium (Biochrom GmbH, Berlin, Germany) and 20% FBS superior (Sigma-Aldrich, St. Louis, MO, USA)) and then transferred to cryovials. After adding 10% dimethyl sulfoxide (DMSO, Carl Roth, Karlsruhe, Germany) dropwise to the suspension, tubes were inverted and transferred into a Mr. Frosty™ freezer container (Thermo Fisher Scientific, Waltham, MA, USA) and stored at −80 °C for at least 24 h. The cells were subsequently transferred to liquid nitrogen for long-term storage.

For the in vitro steatosis experiments, PHHs were seeded with 2 × 10^6^ cells/cm^2^ in 24-well culture plates (Greiner Bio-One International GmbH, Frickenhausen, Germany) which were previously coated with type I collagen that had been prepared from rat tails in our own laboratory according to the protocol of Rajan et al.^36^. PHH adherence was achieved after four hours in PHH culture medium at 37 °C, 5% CO_2_ in a humidified incubator. Following two washes with DPBS at the four-hour mark, steatosis was induced according to the in vitro steatosis model described by Gómez-Lechón et al.^37^. PHH culture medium was replaced by control (Williams MediumE, 100 U/100 µM penicillin/streptomycin, 1 mM sodium pyruvate, 15 mM HEPES, 5% FBS, 1% MEM NEAA supplemented with 0.3% methanol), or FFA-containing medium (control medium supplemented with 0.6 mM oleate/palmitate in a 2:1 ratio (Gibco Invitrogen, Karlsruhe, Germany) in methanol (J.T. Baker, Deventer, The Netherlands)) and hepatocytes were cultured for up to 48 h with a medium change after 24 h.

### Animal maintenance and collection of murine liver samples

Liver tissues were from a previous study published elsewhere^38^ and were of secondary used in accordance with the 3R principles. In brief, liver tissues were collected from normal male C57BL6/J mice (ex-breeder) (Janviers, France) aged 8–9 months with a body weight of 28–30 g (n = 6/group). The animals were maintained in a conventional animal facility under constant environmental conditions, including a twelve-hour light/dark cycle, a constant temperature of 21 ± 2 °C, and a 45–65% relative humidity. Mice were fed for either two or four weeks the same HF-diet (E15652-94 EF R/M, high fat MCD mod, Ssniff Spezialdiäten GmbH, Sulzfeld, Germany) to induce steatosis of different severities whereas the control group of mice received the standard maintenance diet (Altromin Spezialfutter GmbH, Lage, Germany). Food intake and body weight was monitored on a daily basis. Animals were sacrificed using an overdose of isoflurane with exsanguination. Approval for the mouse study was granted by the Thüringer Landesamt für Verbraucherschutz, Thuringia (Approval-Number: UKJ-19–020).

### Operation of the mass spectrometry system and data acquisition

Experiments were carried out on a Xevo G2-XS QTof (Waters, Wilmslow, UK) fitted with a REIMS ion source of the same manufacturer. After the sample evaporation, the aerosol was drawn through an inlet capillary into the source by a Venturi pump through a steady flow of nitrogen gas (standard 20 L/min). The inlet capillary of 20 cm length and 0.7 mm inner diameter exits 5 mm before a Kanthal D metal coil which was operated at a temperature of 150 °C heated by a 3 A DC current at 3.5 V. For the inlet of the leucine enkephalin (Sigma Aldrich, L9133-10MG) standard dissolved in 99.9% isopropanol (LC–MS Grade, Honeywell, Morristown, New Jersey, 34965–2.5 L), a capillary was attached on the housing of the Venturi pump led to a 10 mL syringe (Hamilton, Bonaduz, Schweiz) on a Harvard 11 Elite syringe pump (Holliston, Massachusetts, USA). The flow rate was set to 50 µL/min.

### Heating system (HS)-coupled REIMS analysis

Frozen cell samples were thawed and adjusted to 10^6^ cells/25 µL by resuspension in MilliQ-Water (Reference A+ , Merck, Darmstadt, Germany). Then, 25 µL of the cell suspension was transferred to a borosilicate glass flask manufactured by the glassblowing workshop of Leipzig University, which was preheated on a Mini Hot Plate Preheater MHP30 PD (Miniware, Guangzhou, Yuexiu district, China) to 350 °C. The emerging vapor was guided into the REIMS ion source. Data acquisition was set to negative ion mode on continuum and sensitivity mode with a measuring interval of 0.5 s.

### iKnife-coupled REIMS analysis

Tissue samples were also analyzed using a Xevo G2-XS QTof with a REIMS ion source, which was coupled to an iKnife electrosurgical handpiece and integrated smoke extraction (ERBE No. 20321-028). The iKnife system is a non-FDA approved device used for the purpose of research in an investigational setting and is coupled with a metal electrode (custom made) attached to a neutral electrode (ERBE, Tübingen, Germany, No. 20193-082).

On the day of sampling, the tissue was thawed and placed on the neutral metal electrode on a small piece of paper. To improve conductivity, the paper and sample were wetted with water containing 0.9% NaCl (Sigma Aldrich, St. Louis, Missouri, USA, S3014). For cutting with the monopolar electrosurgical knife, power was set to 25 W and auto-cut mode. A cut lasted around 2 s with about three to six incisions per piece of tissue depending on tissue size. Settings of the mass spectrometer were the same as described above.

### iKnife-coupled REIMS analysis of lipid standards

Lipid Standards were acquired from MERCK (Avanti Polar Lipids, Alabaster, Alabama, USA): Cer (Ceramide) (860519P-5 mg), PA (840857P-25 mg), PI (phosphoinositol) (850142P-100 mg), PG (phosphatidylglycerol) (840457P-25 mg), PE (850757P-25 mg), PS (phosphatidylserine) (840034P-10 mg), PC (850457C-25 mg); Sigma Aldrich (St. Louis, Missouri, USA): GTP (Glycerintripalmitat) (T5888-100 mg), GTO (Glycerintrioleat) (T7140-100 mg)), dissolved in methanol (HPLC-grade, Carl Roth, Karlsruhe, Germany) at 50 °C in a thermomixer (Cell Media, Zeitz, Germany ) and stored at -20 °C. A chicken egg was purchased from a local food retailer the day before use, boiled for 10 min and cut into rectangular shapes of 10 × 5 mm, with a rectangular depression to hold the standard solution. The egg white matrix was prepared for the measurement by bathing it in an isotonic solution with 0.1% formic acid (FA) (A117-50, Fisher Chemical, New Hampshire, USA) for 2 min. The sample was placed on the electrode as described above and 50 µL of the lipid standard (except for ceramide: 25 µL) was applied shortly before measurement at 50 °C. Analysis was done in MassLynx (Version 4.1 SCN909, Waters).

### Data processing and statistical analysis

After acquisition, chromatograms were processed by Massbridge (Version 1.0.29, Waters), which bins and normalizes the spectra, applies an adaptive baseline subtraction and presents the data for further processing in Progenesis Qi. Using this software, peak picking was performed with [M−H]^−^ and [M + Cl]^−^ as adducts, resulting in unbiased raw data. For the in vivo and in vitro data sets, relevant lipid species were identified in ProgenesisQi by a combination of LIPID MAPS® and a mass list generated from literature-based MS data.

A second data set was acquired by p-value filtering of the unbiased data set.

Data matrices generated from both data sets (cohort 1 and 2) with EZinfo (Version 3.0.3.0, Sartorius, Göttingen, Germany) were exported and analyzed using Python (Version 3.9, Python Software Foundation, Delaware, USA). Python was used to visualize the PCA, since it is possible to export data from Progenesis Qi but no images. Further, Python was used for creation of the volcano plots, box plots and Venn diagramms. Used packages were statsmodels(0.13.2), pandas(1.4.4), matplotlib(3.5.2), numpy(1.21.5), scipy(1.9.1), seaborn(0.11.2), scikit-learn(1.0.2), matplotlib_venn(0.11.10). The used scripts are uploaded to github.

## Supplementary Information

Supplementary Information.

## Data Availability

The data sets generated during and/or analyzed during the current study are available at 10.5281/zenodo.14198282. While the Python Code which was implemented for this study is available at https://github.com/ReneHaensel/REIMS_Steatotic_Liver_Tissues-Cells_Analysis.
