# Supplementary material for: Application of rapid evaporative ionization mass spectrometry in preclinical and clinical analyses of steatotic liver tissues and cells
Source: Sci Rep. 2025 Mar 18;15:9226. doi: 10.1038/s41598-025-93305-w (PMC11914079; doi:10.1038/s41598-025-93305-w)
Supplement: Supplementary file 1 — Supplementary Information. [file 41598_2025_93305_MOESM1_ESM.pdf]

Supplementary File

Application of Rapid Evaporative Ionization Mass Spectrometry in Preclinical and Clinical Analyses of Steatotic Liver Tissues and Cells

Julian Connor Eckel[1,2,#], Lena Seidemann[1,2,8,#], Mohamed Albadry[3,4], Gerda Schicht[1,2], Marija Skvoznikova[1,2], Sandra Nickel [1,5,8], René Hänsel[2,6], Daniel Seehofer[1,2,8], Grit Gesine Ruth Hiller[7], Hans-Michael Tautenhahn[1,3,5,8], Uta Dahmen[3], Georg Damm[1,2,8,*]

[1] Department of Hepatobiliary Surgery and Visceral Transplantation, Clinic for Visceral, Transplant, Thoracic and Vascular Surgery, Leipzig University Medical Center, Leipzig, Germany.

[2] Saxonian Incubator for Clinical Translation (SIKT), Leipzig University, Leipzig, Germany.

[3] Department of General, Visceral and Vascular Surgery, Experimental Transplantation Surgery, University Hospital Jena, Jena, Germany.

[4] Department of Pathology, Faculty of Veterinary Medicine, Menoufia University, Shebin Elkom, Menoufia, Egypt

[5] Department of General, Visceral and Vascular Surgery, University Hospital Jena, Jena, Germany

[6] Institute for Medical Informatics, Statistics and Epidemiology (IMISE), Leipzig University, Leipzig, Germany.

[7] Institute for Pathology, University Hospital, Leipzig University, 04103 Leipzig, Germany;
[8] Comprehensive Cancer Center Central Germany (CCCG), Jena and Leipzig, Germany

[#] Both authors contributed equally to this work and are considered shared first authors.

[*] Correspondence: georg.damm@medizin.uni-leipzig.de

A

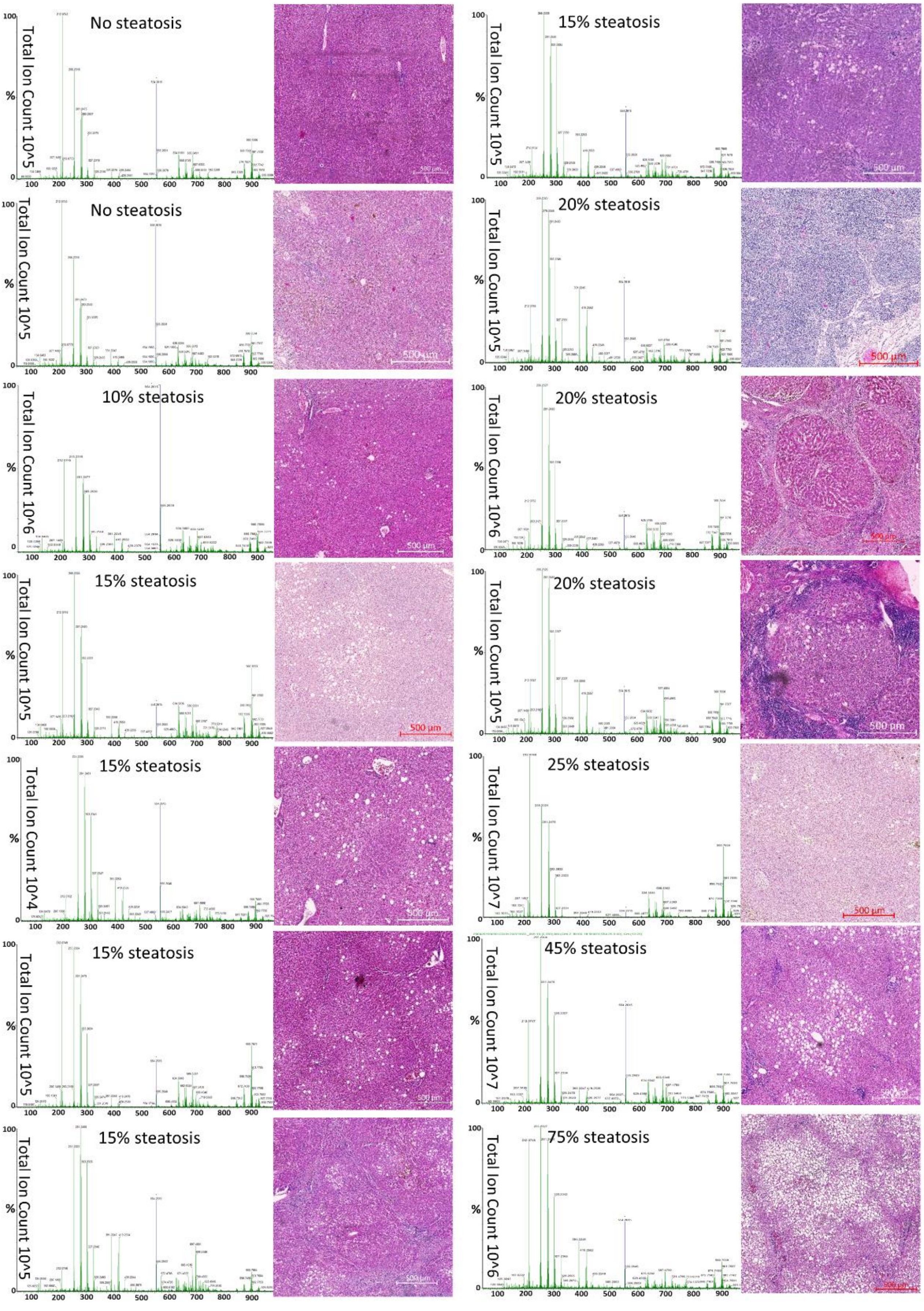
No steatosis
No steatosis
10% steatosis
15% steatosis
15% steatosis
15% steatosis
15% steatosis
15% steatosis
20% steatosis
20% steatosis
20% steatosis
25% steatosis
45% steatosis
75% steatosis
Total Ion Count 10^5
Total Ion Count 10^6
Total Ion Count 10^4
Total Ion Count 10^7
%
500 µm

24

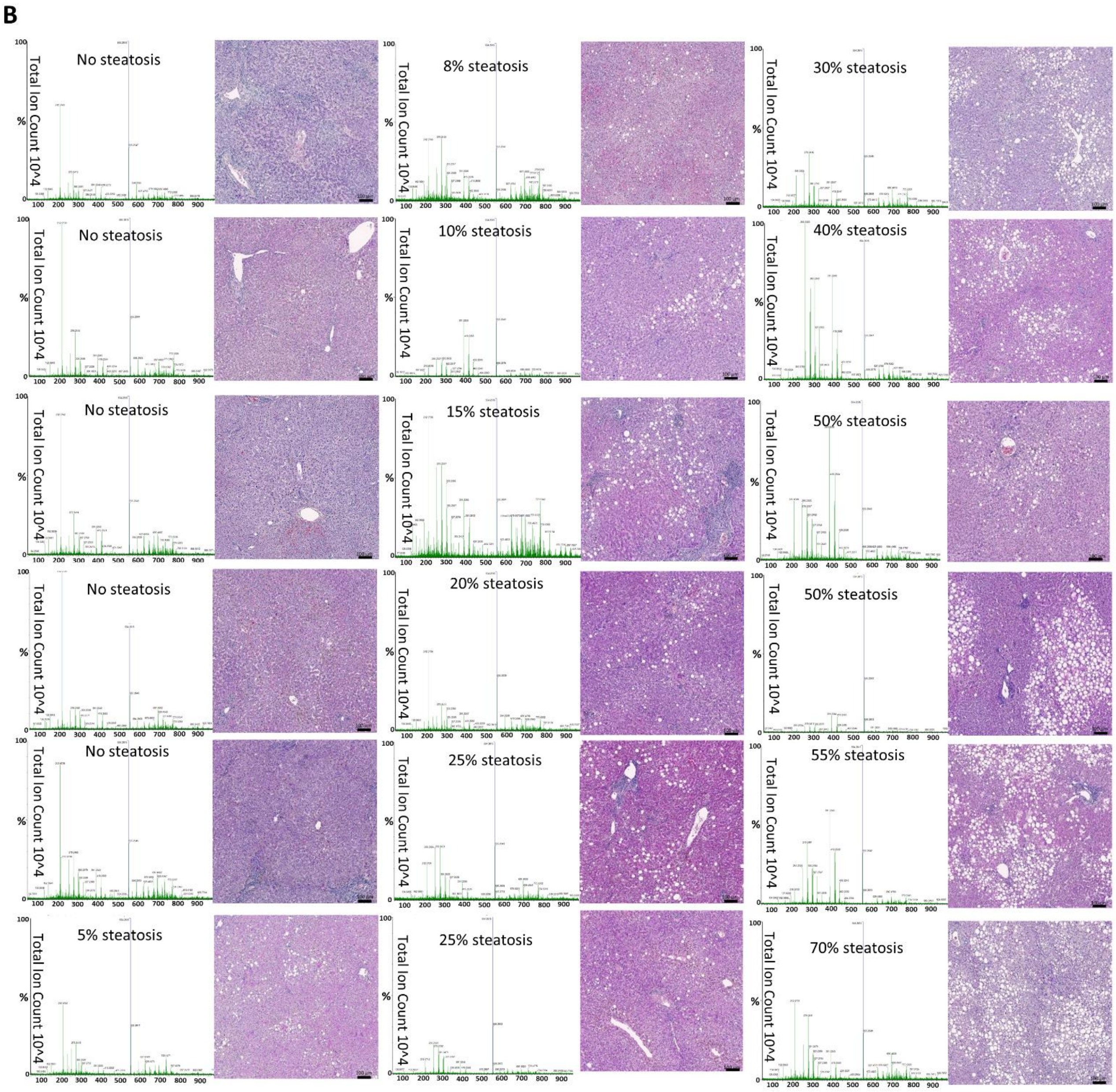

Supplementary Figure 1 REIMS spectra and pathological valuation of the lipid content in human liver tissue. Human liver tissues were stained with H/E and lipid content was determined by a pathologist. Respective mass spectra from REIMS analysis of the same liver tissues are shown on the left side of the microscopy images. **(A)** Cohort 2 **(B)** Cohort 1.

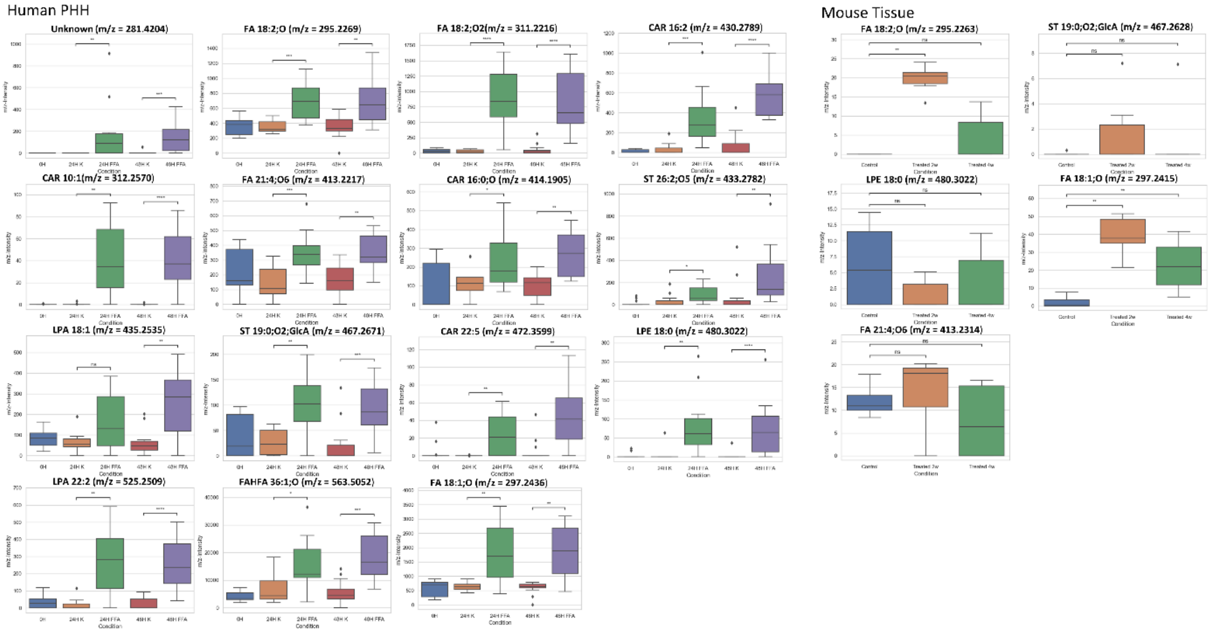

Supplementary Figure 2 Investigation of lipids relevant for steatosis development in vitro and in vivo. (A) Median/quartile minimum/maximum box plots of selected lipids from (Fig. 4A). **(B)** Median/quartile minimum/maximum box plots of selected lipids from (Fig. 4C). ns: not significant, *: $p < 0.05$, **: $p < 0.01$, ***: $p < 0.001$, ****: $p < 0.0001$; Kruskal-Wallis ANOVA-type test. The assignment of identifications to determined m/z was carried out using LipidMAPS.

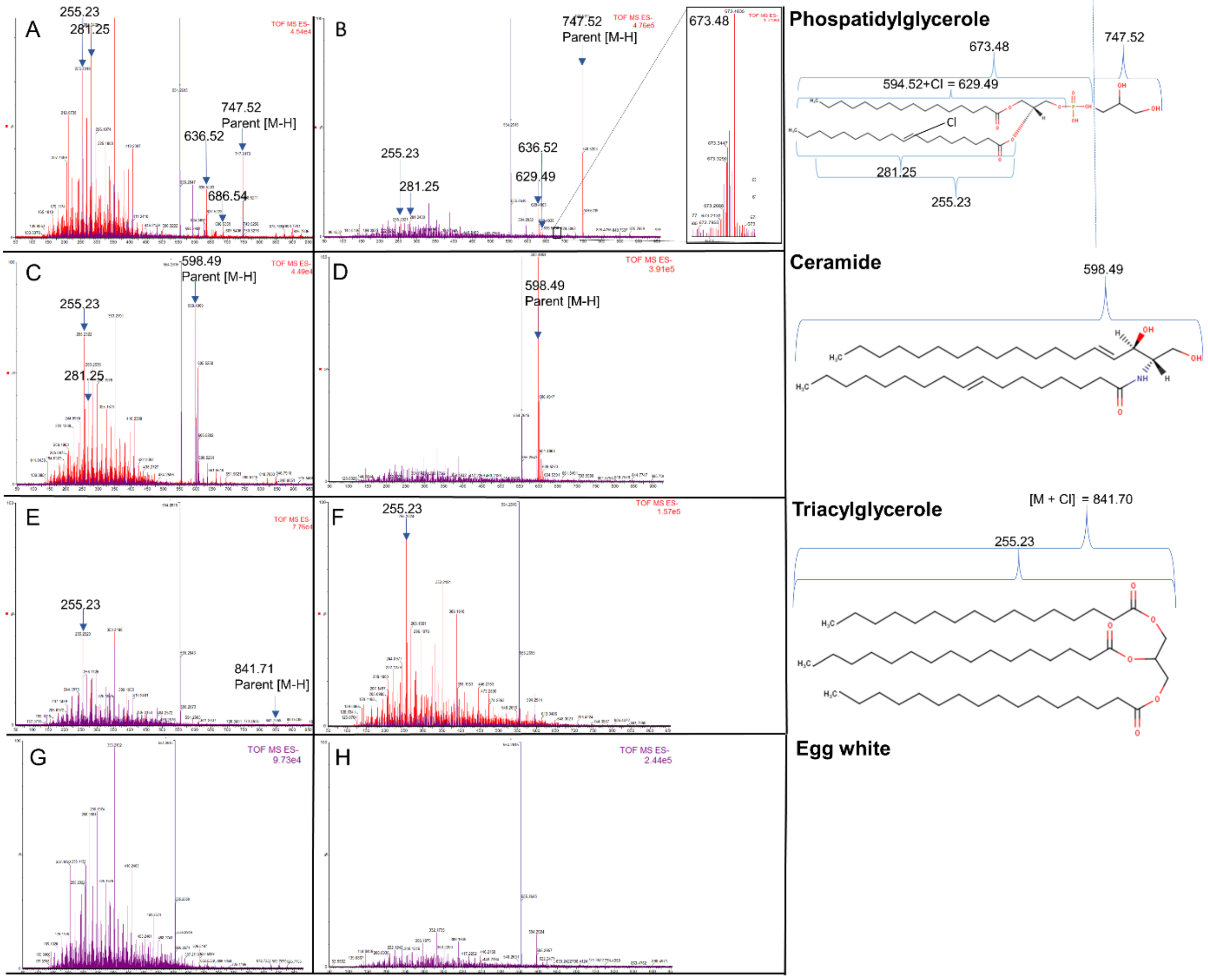

Supplementary Figure 3 iKnife-coupled REIMS of the last lipid standards. On the left side **(A, C, E)** saline solution treated egg white standards are presented. While on the right side samples treated with saline solution and 0.1% formic acid are shown **(B, D, F)**. Parent masses and fragments of interest are highlighted. **(G-H)** show examples of the egg white matrix withour applied standard on the left side treated with saline solution and on the right side with a mixture of saline and formic acid.

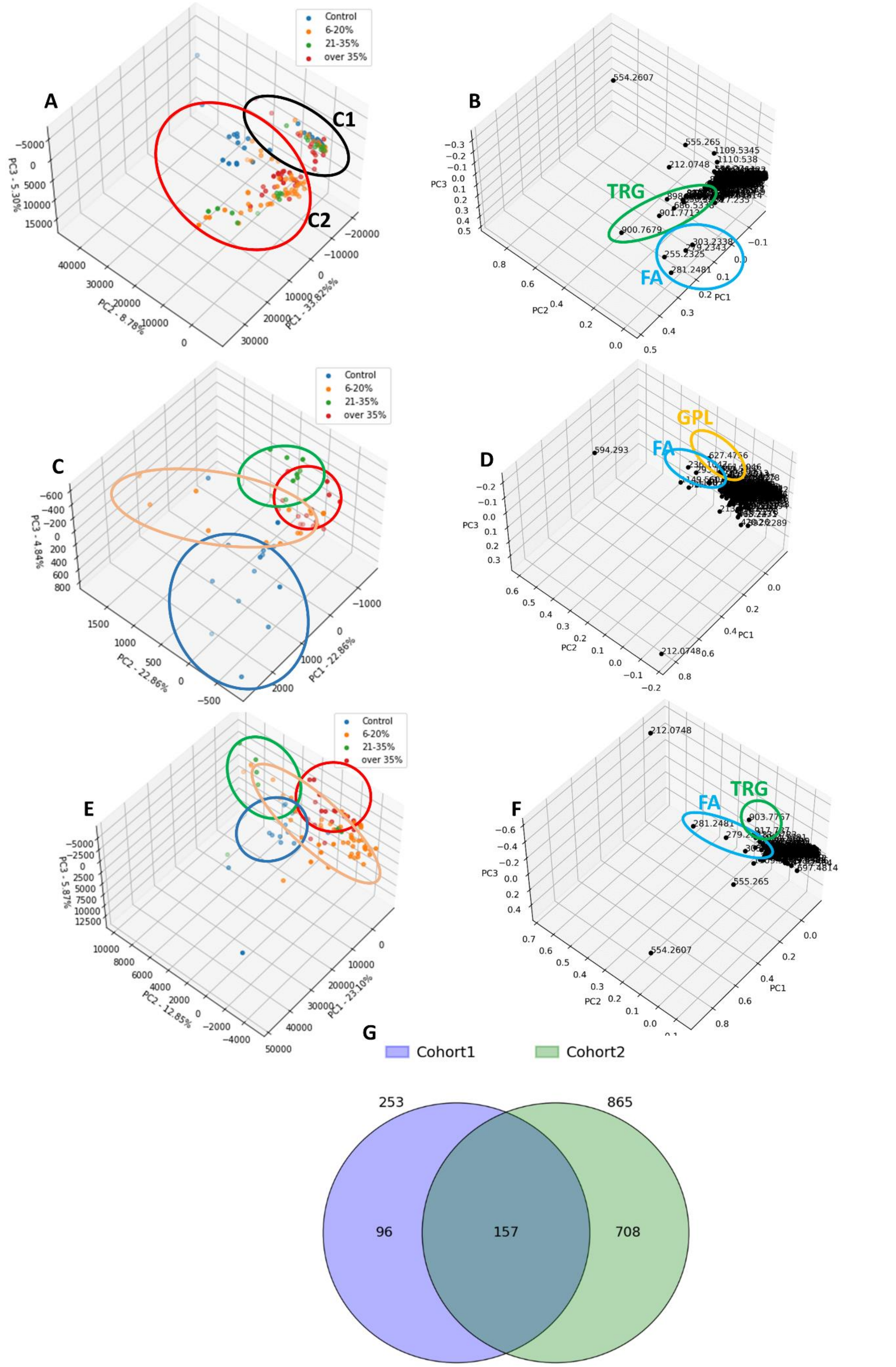

Supplementary Figure 4: Extended data analysis of REIMS data. Data from Fig. 2 were assigned the individual samples to conditions according to their clinically reported severity of

steatosis (control (<6%), 6-20%, 21-35%, >35%), filtered with a p-value ≥ 0.05 and reduced to a mass range of m/z 250 to 1,000. PCA of the combined data **(A)** and of the separated cohorts according to tissue source **(C, E)** Loadings of the combined data **(B)** and the separate cohorts **(D, F)**. The loadings consist of 253 masses for cohort 1 and 865 masses for cohort 2; FA: fatty acids, TAG: triacylglycerides, GPL; glycerophospholipids. (G) shows the overlap of masses determined in both cohorts.

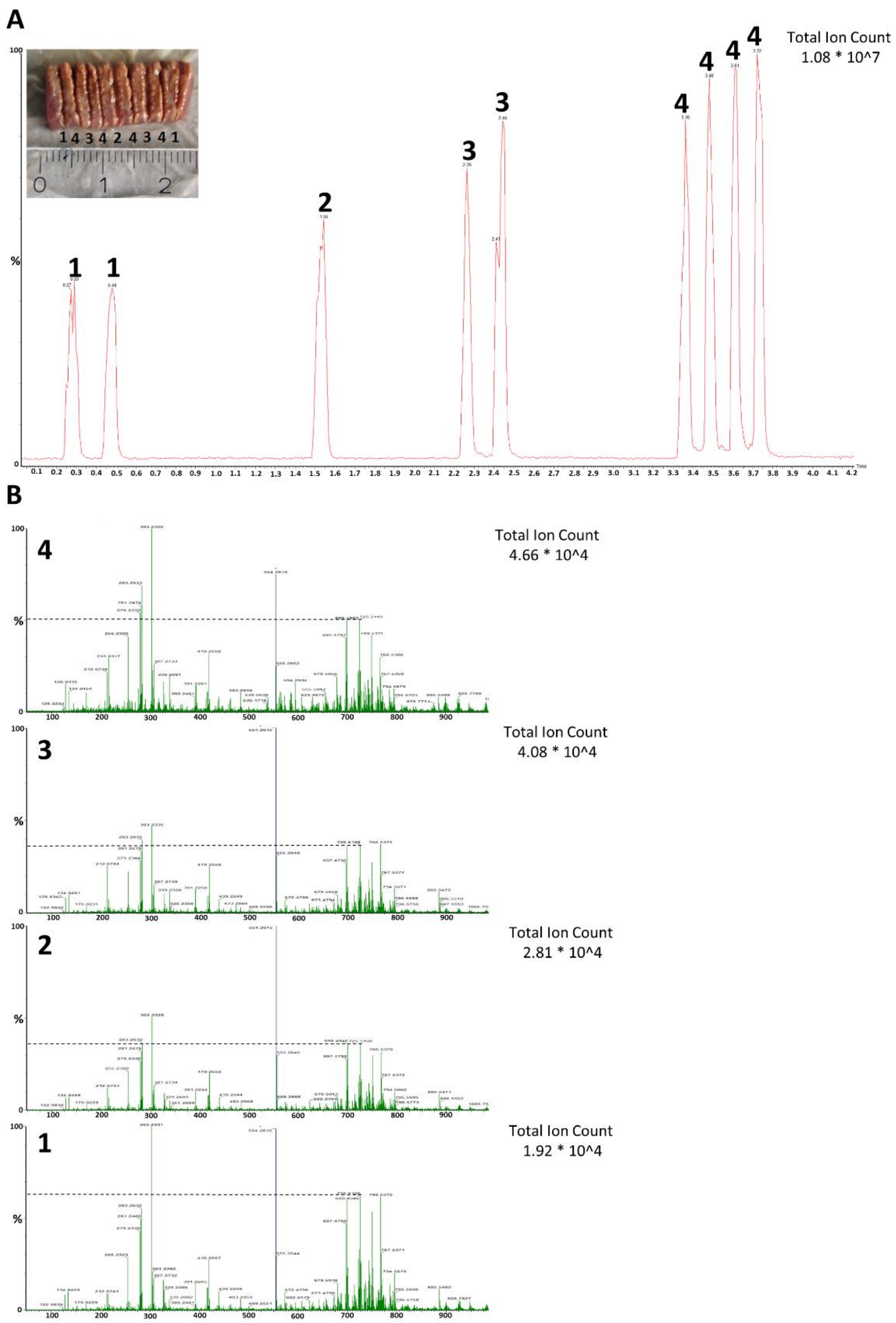

Supplementary Figure 5 Influence of the distance between two cuts on the lipid fingerprint. A human liver tissue sample was cut with maximum distance between two cuts. For further cuts the distance was halved resulting in a decreasing distance with an increasing number of cuts. **(A)** The tissue used for REIMS analysis is shown together with the resulting chromatogram. Each peak represents a cut with the iKnife in the human tissue sample **(B)** Representative mass spectra of peaks from the chromatogram shown in (A). The dashed line marks the highest m/z value measured in the glycerophospholipid cluster. With decreasing

cutting distance the relative abundance of the GPL cluster decreases, while the total ion count increases.

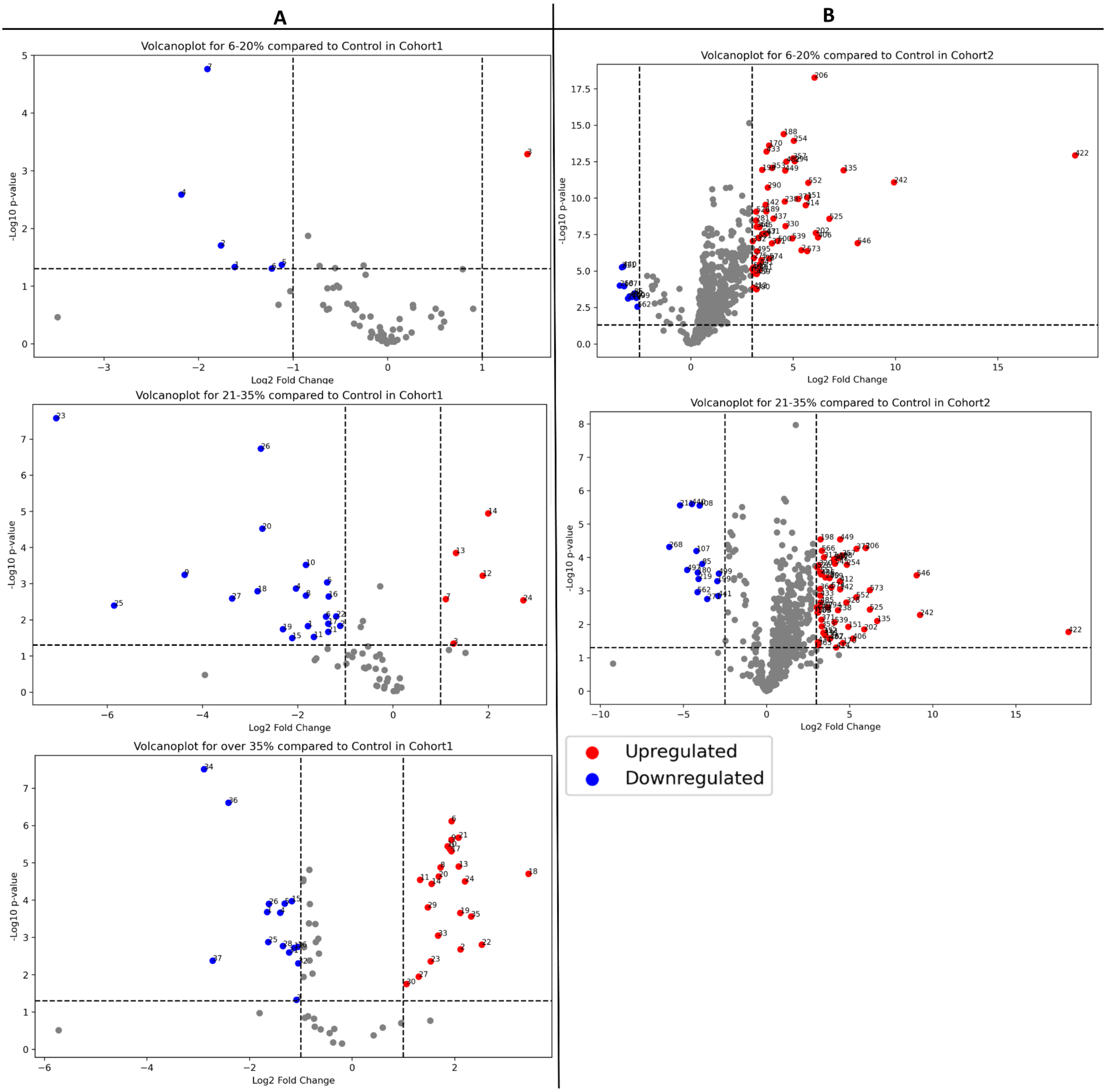

Supplementary Figure 6 Extended view on data reduction. Shown are comparisons of further steatosis groups in comparison to control of both cohorts by unpaired, two-sided t-test. Significantly increased and decreased masses with a log2 fold change for **(A)** cohort 1 of < -1 or > 1 and **(B)** cohort 2 of < -2.5 or > 3 were defined as relevant. In general, cohort 2 shows a much higher data density than cohort 1.

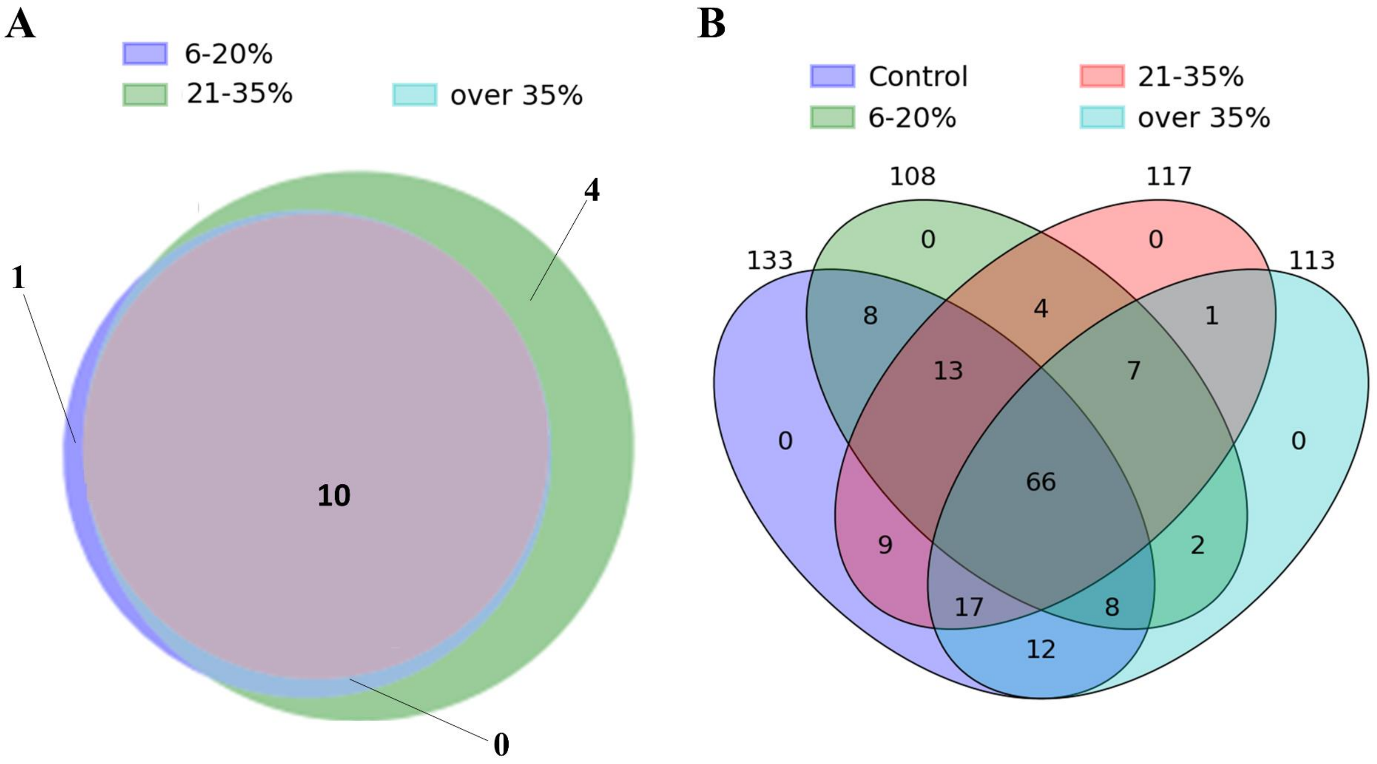

Supplementary Figure 7 Investigation of marker lipids defining clinically reported severity of steatosis. (A) Decreased masses of cohort 2 in comparison to control, complementary to Fig. 7B. The conditions show an overlap in 10 masses, while 5 masses are specific to two groups of steatosis severity. (**B)** All groups of steatosis severity are compared to each other. The overlap shows that no specific mass is able to define a condition on its own.

Cohort 2

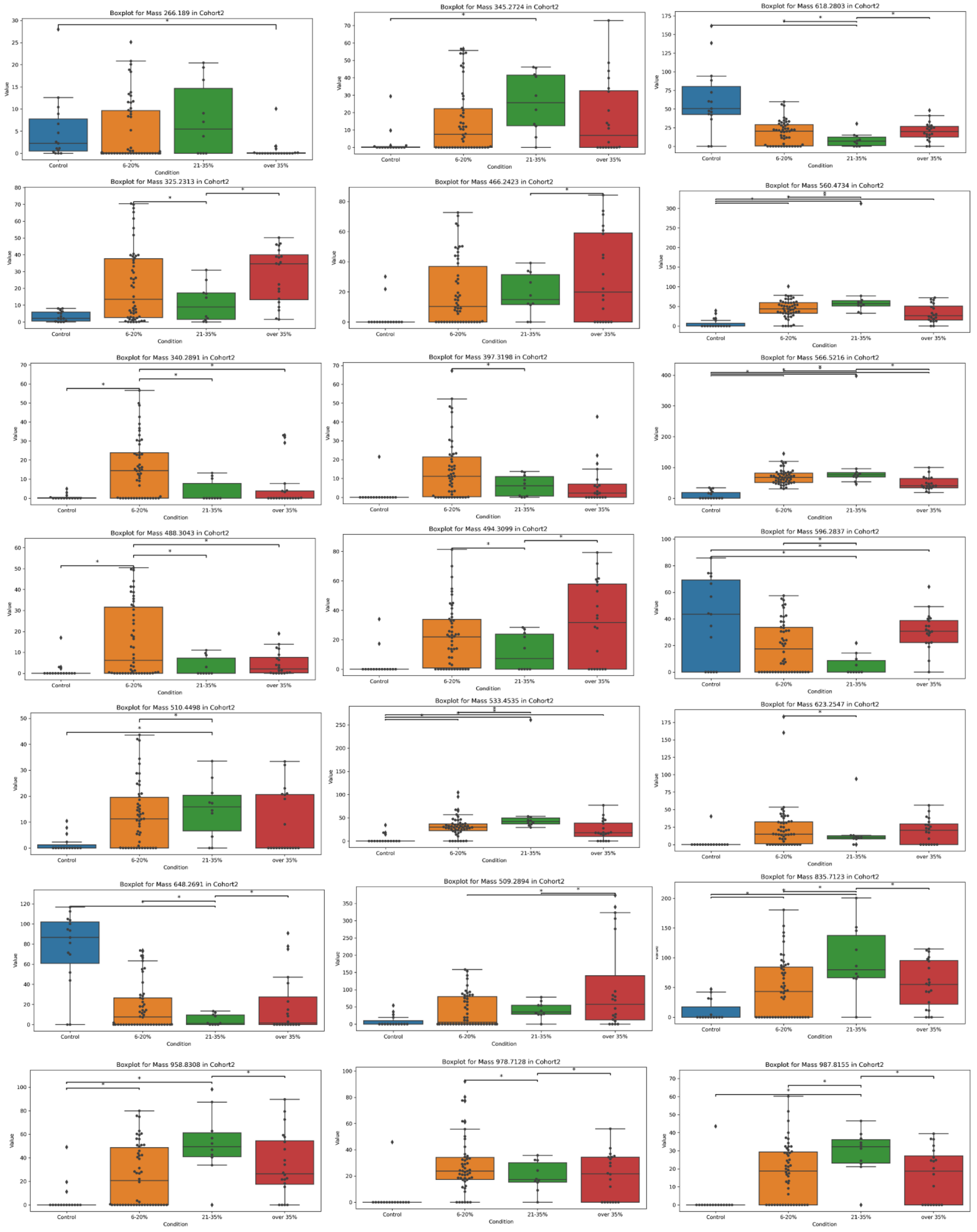
Boxplot for Mass 266.189 in Cohort2
Boxplot for Mass 345.2724 in Cohort2
Boxplot for Mass 618.2803 in Cohort2
Boxplot for Mass 325.2313 in Cohort2
Boxplot for Mass 466.2423 in Cohort2
Boxplot for Mass 560.4734 in Cohort2
Boxplot for Mass 340.2891 in Cohort2
Boxplot for Mass 397.3198 in Cohort2
Boxplot for Mass 566.5216 in Cohort2
Boxplot for Mass 488.3043 in Cohort2
Boxplot for Mass 494.3099 in Cohort2
Boxplot for Mass 596.2837 in Cohort2
Boxplot for Mass 510.4498 in Cohort2
Boxplot for Mass 533.4535 in Cohort2
Boxplot for Mass 623.2547 in Cohort2
Boxplot for Mass 648.2691 in Cohort2
Boxplot for Mass 509.2894 in Cohort2
Boxplot for Mass 835.7123 in Cohort2
Boxplot for Mass 958.8308 in Cohort2
Boxplot for Mass 978.7128 in Cohort2
Boxplot for Mass 987.8155 in Cohort2
Value
Condition
Control
6-20%
21-35%
over 35%

Supplementary Figure 8 extended boxplots of the optimized protocol. Median/quartile minimum/maximum box plots of metabolite masses used for discrimination of the conditions in cohort 2. (ns: not significant, *: p <= 0.05)

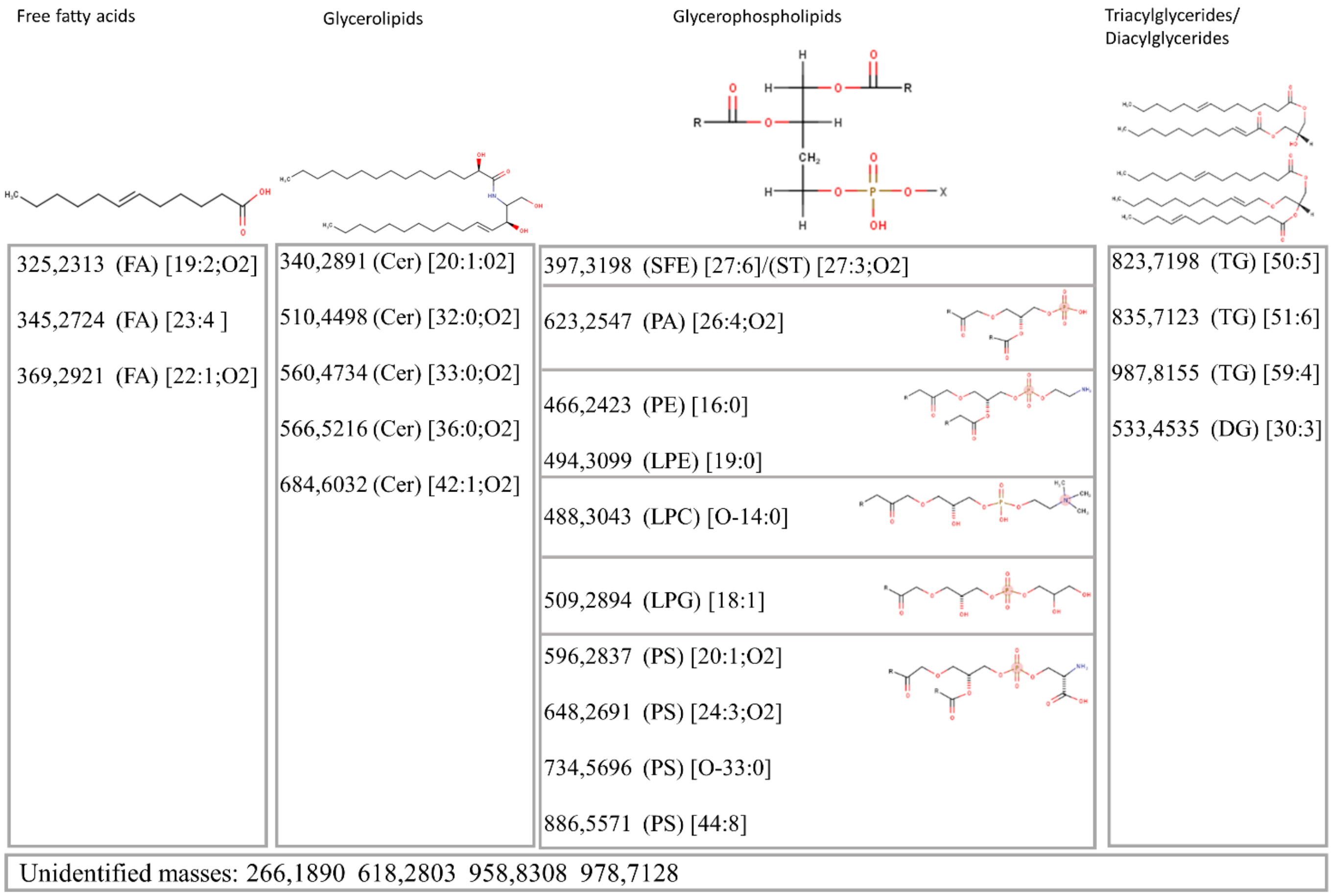

Supplementary Figure 9 Compound identification of influential masses. All 26 masses influential in discriminating the steatotic conditions are shown. Compounds which could be identified by LIPID MAPS® were assigned a lipid species with their tentative sum formula. When two different sum formulas were provided for one compound, both are indicated. SFE = short fatty esters, ST = sterol cholesterol.

Supplementary Table 1: Levene´s test for Cluster Variance analysis. Table 1 presents a comparison between the clusters identified in our visualisation of the data and the correct assigned data points to their respective cohorts. The data points were visualized using PCA and represented in a three-dimensional space. To check the homogeneity of variances between the groups, we conducted the Levene's test for PC1 as it is explaining the main part of the variances. We also calculated the inverse F-value as comparison for the Levene´s number.

	Cluster 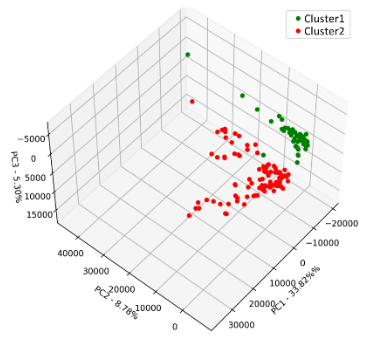	Cohort 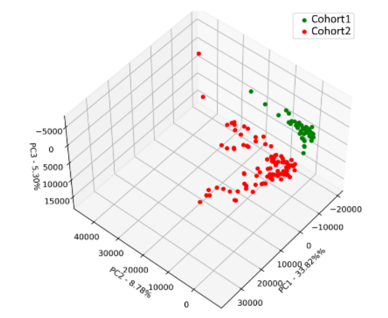
**Levene´s test**		
Inverse F-value	1,468	1,470
Levene´s number (PC1)	45.187	45.562
P-value	3.688e-10	3.186e-10
